# Unsolved Issues in the Management of High Blood Pressure in Acute Ischemic Stroke

**DOI:** 10.1155/2013/349782

**Published:** 2013-04-23

**Authors:** Gordian J. Hubert, Peter Müller-Barna, Roman L. Haberl

**Affiliations:** Department of Neurology and Neurological Intensive Medicine, Klinikum Harlaching, Städtisches Klinikum München GmbH, Thalkirchner Straße 48, 80337 Munich, Germany

## Abstract

High blood pressure is common in acute stroke patients. Very high as well as very low blood pressure is associated with poor outcome. Spontaneous fall of blood pressure within the first few days after stroke was associated both with neurological improvement and impairment. Several randomized trials investigated the pharmacological reduction of blood pressure versus control. Most trials showed no significant difference in their primary outcome apart from the INWEST trial which found an increase of poor outcome when giving intravenous nimodipine. Nevertheless, useful information can be extracted from the published data to help guide the clinician's decision. Blood pressure should only be lowered when it is clearly elevated, and early after onset, reduction should be moderate but may be achieved rapidly. No clear recommendations can be given on the substances to use; however, care should be taken with intravenous calcium channel blockers and angiotensin receptor antagonists. Two ongoing randomized trials will help to solve the questions on blood pressure management in acute stroke.

## 1. Introduction

High blood pressure occurs in around 80% of patients with acute ischemic stroke [1] and usually decreases over the following 7 days [2]. 

Whether this elevated blood pressure is a physiological and protective reaction or a side effect of a generalised stress reaction that is in fact harmful to the newly damaged brain and whether it should be lowered or not is yet to be answered. This problem is considered as “one of the major unresolved issues in acute stroke management” [3]. American (AHA) and European (ESO) guidelines recommend not to treat elevated blood pressure unless above 220/120 mmHg and describe an urgent need for large well-designed trials to address this issue [4, 5]. Currently there are two large ongoing trials: the “Efficacy of Nitric Oxide in Stroke” trial (ENOS) compares nitric oxide versus placebo and continuing versus stopping previously taken antihypertensive agents in acute stroke [6]. ENCHANTED compares low dose versus normal dose of recombinant tissue-type plasminogen activator (r-tPA) and intensive blood pressure reduction versus standard reduction in thrombolysed patients [7]. 

This article focuses on currently available data, the contraries of the results, and potential solutions. 

## 2. Initial Blood Pressure and Outcome

Several observational studies looked into the relationship of high initial blood pressure and outcome. A post hoc analysis of the International Stroke Trial found a U-shaped relationship between initial blood pressure and outcome, indicating that systolic blood pressure of around 140–180 mmHg is associated with best outcome [1]. These findings were confirmed in another study showing that very high and very low initial blood pressure is associated with increased death rates [8]. High blood pressure was also independently associated with recurrent ischemic stroke and cerebral edema [1, 9].

Patients with very low initial blood pressure had a higher incidence of heart failure and coronary heart disease. This might explain part of their worse outcome. The reason for poor outcome after initial high blood pressure seems more complex. High blood pressure after acute ischemic stroke causes brain edema [10], and brain edema decreases cerebral blood flow [11, 12], thus aggravating cerebral ischemia. Another mechanism could be that endogenous tPA release is reduced when blood pressure is high due to the damage at the endothelium [13–15].

One critique of those studies is that blood pressure was measured only on admission, usually several hours after stroke onset [1, 8, 16, 17]. However, variability and duration of high blood pressure in the first hours after stroke onset may be more relevant for prognosis.

## 3. Spontaneous Change in Blood Pressure and Outcome

Several observational studies looked into the prognosis of spontaneous change in blood pressure during the acute phase with divergent results. The Barcelona Downtown stroke registry found that early decrease of systolic blood pressure by 20–30% was associated with full recovery (OR 2.9, 95% CI 1.3–6.3) [16]. A decrease in systolic blood pressure within 12 hours was associated with recanalization of the vessel [18]. In other studies spontaneous decrease of blood pressure within 24 hours was associated with poor outcome [19] and early neurological deterioration [20]. 

There might be a bias when looking into spontaneous decrease of blood pressure and correlating it to outcome. Rapid spontaneous decrease might reflect less severe stroke [17], recanalization of the vessel [18], or might be due to cardiac insufficiency [8], all of which have an independent effect on outcome. So it might be a sign for a rapidly resolving or rapidly deteriorating process, explaining the divergent results.

## 4. Intervention of Elevated Blood Pressure and Outcome

Several randomised controlled trials addressed the topic of blood pressure management in acute stroke (see Table 1). The trials are heterogeneous regarding time to treatment, degree and speed of effective blood pressure reduction, substances used, and the stroke subgroups included (i.e., ischemic, hemorrhagic strokes, or both). 

*Beta-blockers* given within 48 hours after hemispheric stroke increased mortality (BEST) [21]. The treatment groups, however, were unbalanced with more severely disabled patients in the group receiving beta blockers. Also patients did not have to have elevated blood pressure to be included in the trial. The calcium antagonist *nimodipine* was investigated in several trials in the 1990s. A metaanalysis of nine trials showed a significant benefit from oral nimodipine versus placebo when given within 12 hours of symptom onset [22]. In contrast, intravenous nimodipine given as 1 mg/h and 2 mg/h increased the rate of poor neurological and functional outcome in ischemic stroke patients compared to placebo (INWEST) [23]. A correlation was found between the amount of diastolic blood pressure reduction in the high-dose group and poor functional outcome [24]. 

Intravenous *magnesium* administered within 12 hours lowered systolic blood pressure by 4 mmHg and showed a trend towards improved outcome in all stroke patients (odds ratio: 0.95, 95% CI 0.80–1.13, *P* = 0.59). For the subgroup of patients with elevated blood pressure (mean arterial pressure >108 mmHg), improvement was significant (odds ratio of 0.78, 95% CI 0.61–0.99) [25]. 

Smaller trials with *glyceryl trinitrate* showed no significant difference in functional outcome [26, 27] but were also not powered to do so.

The early administration of *labetalol* and *lisinopril* lead to a significant reduction in blood pressure as compared to the placebo group (mean systolic difference 10 mmHg, 95% CI 3–17, *P* = 0.004) (CHHIPS). Death and dependency at 3 months did not differ, but 3 months mortality was halved in the treatment group to 9,7% from 20,3% (HR 0.4, 95% CI 0.2–1.0, *P* = 0.05). The intervention was safe regarding early neurological outcome and serious adverse events [28].

The COSSACS trial looked into the question whether previously taken antihypertensive agents should be stopped or continued in patients with acute stroke. The trial included patients within 48 hours from symptom onset. Continuing the medication reduced blood pressure significantly (13/8 mmHg lower at two weeks). No difference was seen in the primary outcome death or dependency at two weeks, but there was a significant functional improvement in the subgroup of patients with confirmed acute ischemic stroke (relative risk 0.70, 95% CI 0.51–0.99, *P* = 0.045) [29].

Another trial (SCAST) compared *candesartan* to placebo within 30 hours after onset looking at a combined vascular endpoint. Blood pressure reduction was only moderate, and no significant differences in the endpoints were seen. A trend favouring placebo was noticed [30].

A metaregression on actively lowered blood pressure in acute stroke including 9008 patients from randomized trials showed the lowest odds ratio (0.95, 95% CI 0.11–1.72) for death or dependency with a decrease of systolic blood pressure by 14 mmHg [31]. 

Two Cochrane analyses concluded that there is insufficient evidence to evaluate the effect of blood pressure changes on outcome after acute stroke [32, 33].

## 5. Discussion

The management of blood pressure in acute ischemic stroke remains unclear due to divergent results of the studies. The effect of blood pressure management appears to differ in various patient populations. The effect might be dependent on level of initial blood pressure, time to treatment, stroke severity, and history of hypertension, as well as intensity of blood pressure lowering, substances used, and administration form (e.g., i.v. or oral). Some of the possible influences should be looked at in more detail.

### 5.1. Initial Level of Blood Pressure

It is evident that lowering blood pressure of normotensive patients in acute stroke may lead to hypotensive events, thus deteriorating outcome. Therefore, blood pressure lowering should only be considered when it is elevated. The negative effect of the BEST trial might partly be explained by inclusion of normotensive patients with systolic blood pressure >100 mmHg [21]. In a subgroup of IMAGES only those patients with higher blood pressure levels had a benefit (mean arterial pressure >108 mmHg) [25]. This matches with CHHIPS and COSSACS in which mean systolic level was high (181 mmHg and 150 mmHg), and decreased mortality and improved functional outcome in the subgroup with ischemic stroke were seen, respectively [28, 29]. Observational data showed that low initial blood pressure increased poor outcome [1, 8]. However, there are also trials in which treating elevated blood pressure did harm (INWEST) or showed a trend favoring placebo (SCAST) [23, 30]. 

### 5.2. When to Start Treatment

The earlier acute stroke treatment is initiated, the better the outcome [34]. This seems to apply to blood pressure management too. Significant benefit from oral nimodipine versus placebo was seen when given within 12 hours after onset, no benefit between 12 and 24 hours, and worse outcome when initiated after 24 hours [22]. In IMAGES, magnesium was given early at a mean of about 7 hours after onset (max. 12 hours) and showed a benefit in those with elevated blood pressure [25]. In SCAST, treatment was started after about 17 hours and did no benefit. A small subgroup treated within 6 hours, however, did better [30]. In fact, the later treatment was started, the more did Candesartan seem to harm the patient regarding the composite vascular endpoint [30]. Other trials showed the opposite. Nimodipine was started early (11 hours after onset) and had a negative effect [23]. CHHIPS and COSSACS started treatment late and were neutral, even with some positive secondary endpoints [28, 29]. Although the start of treatment is known for most of the trials, the lag until achieving effective blood pressure reduction is not. This may influence the results.

### 5.3. How Fast and How Much to Lower Blood Pressure

It is agreed that a rapid and large decrease in blood pressure is dangerous for acute stroke patients. This opinion is largely based on the data from INWEST and some observational data [19, 20, 23]. High-dose (but not low-dose) intravenous nimodipine was associated with death and dependency when diastolic blood pressure was lowered by >20% within the first two days [24]. A secondary analysis of SCAST showed that a large systolic decrease of >28 mmHg was significantly associated with poor outcome [35]. Moderate reduction of blood pressure seems to be safe and even protective. Magnesium reduced blood pressure within 24 hours by 4/3 mmHg and showed a benefit for those with higher blood pressure. In CHHIPS the systolic blood pressure difference was 10 mmHg within the first 24 hours, and mortality was halved after 3 months. A decrease of even 16 mmHg was achieved within 4 hours with labetalol compared to placebo, and this was found to be safe [28]. So the rapid reduction does not seem to be dangerous, as long as reduction is moderate. 

### 5.4. Which Substance to Use

No clear answer can be given to this question as yet. Some trials included various classes [28, 29], and others looked into the effect of a single one. The calcium channel blocker nimodipine had a positive effect when given orally within 12 hours, but a negative effect when given intravenously. The angiotensin-converting-enzyme inhibitor lisinopril had a positive effect on 3 months mortality [28], and the angiotensin receptor antagonist candesartan possibly has an injurious effect on the brain [30]. Nitric oxide donors found stable cerebral blood flow albeit reducing blood pressure effectively [27, 36], and the mixed alpha/beta-receptor antagonist labetalol showed reduction of 3-month mortality [28].

### 5.5. Safety

Several data showed that lowering blood pressure in acute ischemic stroke is safe in terms of early neurological deterioration and functional outcome [26–29], even in the presence of carotid artery stenosis [37] and that it does not decrease perfusion in the region of the infarct [27, 36, 38–40].

## 6. Conclusion

Trials looking at the effect of blood pressure reduction in the acute phase of stroke are heterogeneous in design, classes of antihypertensive agents, achieved blood pressure reduction, and influence on outcome. Although the guideline recommendation is not to lower blood pressure in the range up to 220/120 mmHg, there might be some neuroprotective effect of very early (first 7–10 hours after stroke) and rather moderate (10–20 mmHg) blood pressure lowering. Substance classes may differ in that respect, but care should be taken with intravenous calcium channel blockers and angiotensin receptor antagonists. Adequately powered, randomized trials, however, are urgently warranted to substantiate those speculations.

## Figures and Tables

**Table 1:** Important randomised controlled trials of intervention versus control. SBP: systolic blood pressure, MAP: mean arterial pressure, AH: antihypertensive agents, p.o.: per os, i.v.: intravenous, n.p.: not published. (The phase II trial ACCESS comparing candesartan versus placebo has not been included, as the following phase III trial SCAST has been).

Name	Year of publication	*n*	Initial median blood pressure (mmHg)	Time to treatment (hours)	Substance	Administration	Stroke subgroup
BEST [21]	1988	302	n.p.	22–25.3	Atenolol, Propanolol	p.o.	Ischemic + hemorrhagic
INWEST [23]	1994	295	SBP 159–161	10.5–11.5	Nimodipine	i.v.	Ischemic
Rashid et al. [27]	2003	90	SPB 151	51	Glyceryl Trinitrate	transdermal	Ischemic + hemorrhagic
IMAGES [25]	2004	2589	MAP 108	7	Magnesium	i.v.	Ischemic + hemorrhagic
CHHIPS [28]	2009	179	SBP 181	17.4–20.5	Labetalol, Lisinopril	p.o., i.v., sublingual	Ischemic + hemorrhagic
COSSACS [29]	2010	763	SBP 150	23.5	Previously taken AH	any	Ischemic + hemorrhagic
SCAST [30]	2011	2029	SBP 171	17.8	Candesartan	p.o.	Ischemic + hemorrhagic
